# Translatable mitochondria-targeted protection against programmed cardiovascular dysfunction

**DOI:** 10.1126/sciadv.abb1929

**Published:** 2020-08-19

**Authors:** K. J. Botting, K. L. Skeffington, Y. Niu, B. J. Allison, K. L. Brain, N. Itani, C. Beck, A. Logan, A. J. Murray, M. P. Murphy, D. A. Giussani

**Affiliations:** 1 Department of Physiology, Development and Neuroscience, University of Cambridge, Downing Street, Cambridge CB2 3EG, UK.; 2 Cambridge Cardiovascular Strategic Research Initiative, Cambridge, UK.; 3 Barcroft Centre, University of Cambridge, Cambridge, UK.; 4 MRC-Mitochondrial Biology Unit, University of Cambridge, Hills Road, Cambridge CB2 0XY, UK.; 5 Cambridge Strategic Research Initiative in Reproduction, Cambridge, UK.

## Abstract

The prenatal origins of heart disease in offspring have been established. However, research in species with developmental milestones comparable to humans is lacking, preventing translation of this knowledge to clinical contexts. Using sheep and chickens, two species with similar cardiovascular developmental milestones to humans, we combined in vivo experiments with in vitro studies at organ, cellular, mitochondrial, and molecular levels. We tested mitochondria-targeted antioxidant intervention with MitoQ against cardiovascular dysfunction programmed by developmental hypoxia, a common complication in human pregnancy. Experiments in sheep determined in vivo fetal and adult cardiovascular function through surgical techniques not possible in humans, while those in chicken embryos isolated effects independent of maternal or placental influences. We show that hypoxia generates mitochondria-derived oxidative stress during cardiovascular development, programming endothelial dysfunction and hypertension in adult offspring. MitoQ treatment during hypoxic development protects against this cardiovascular risk via enhanced nitric oxide signaling, offering a plausible intervention strategy.

## Teaser

Mitochondrial therapy may cure hypertension in adult offspring of complicated pregnancies.

## INTRODUCTION

Despite improved understanding and advances in treatment, cardiovascular disease still claims the life of one in three people and costs the United States and Canada US$130 billion and the United Kingdom over £30 billion every year (*1*, *2*). Most of these costs are attributed to treatments that improve outcomes but do not cure the disease. For this reason, greater focus has been placed on prevention. In addition to the interaction between genes and lifestyle risk factors, such as smoking and obesity, the gene–early environment interaction during pregnancy also plays a role in determining heart disease risk in the adult offspring—a process known as developmental programming (*3*–*5*). For instance, children born to the same mother after bariatric surgery to control obesity had lower rates of insulin resistance and high blood pressure compared to those born before (*6*, *7*). Such studies in siblings highlight a disproportionate risk of disease in offspring gestated in the same womb but under different in utero conditions, providing robust evidence in humans that the environment experienced during sensitive periods of development directly influences long-term cardiovascular health.

In humans, a common complication of pregnancy is a reduction in fetal oxygenation (chronic fetal hypoxia), which leads to fetal growth restriction (*8*). Experiments in rodent animal models have established that chronic fetal hypoxia programs an increased risk of cardiovascular disease in the adult offspring and that a major mechanism involved is increased oxidative stress on the fetal heart and circulation (*5*, *9*, *10*). However, the human clinical relevance of these studies is difficult to prove. When working with animal models of cardiovascular dysfunction before birth, the temporal profile of cardiovascular development between species is a highly important consideration for successful translation to the human clinical situation. Rats and mice are altricial species, born with a highly immature cardiovascular system. Rodents also give birth to litters, so differences in maternal metabolic adaptations to pregnancy in highly polytocous species require clear thought. In contrast, sheep and humans share similar prenatal tempos of precocial cardiovascular development, and some breeds of sheep, like Welsh Mountain, give birth primarily to singleton lambs of similar weight to term human babies (*11*). A recent study in Welsh Mountain sheep showed that maternal treatment with the antioxidant vitamin C in hypoxic pregnancy prevented fetal oxidative stress, fetal growth restriction, and hypertension in the adult offspring (*12*). While this study provides proof of principle that maternal antioxidant therapy in hypoxic pregnancy is protective, only high doses of vitamin C incompatible with human treatment were effective (*12*). In addition, we have reported that an increase in the fetal peripheral vascular oxidant tone, generated by an increased ratio of superoxide anion (O_2_^•−^) relative to nitric oxide (NO) production, is an important physiological component of the fetal brain–sparing response, which redistributes blood flow away from peripheral circulations toward the fetal brain during periods of acute fetal stress (*8*). This effect is problematic because fetal exposure to vitamin C increases NO bioavailability and thus potentially weakens fetal brain sparing by opposing peripheral vasoconstriction (*13*). Therefore, there is an urgent need for alternative antioxidant therapy in doses compatible with human treatment and via mechanisms that maintain fetal brain–sparing circulatory defenses.

**Fig. 1:**
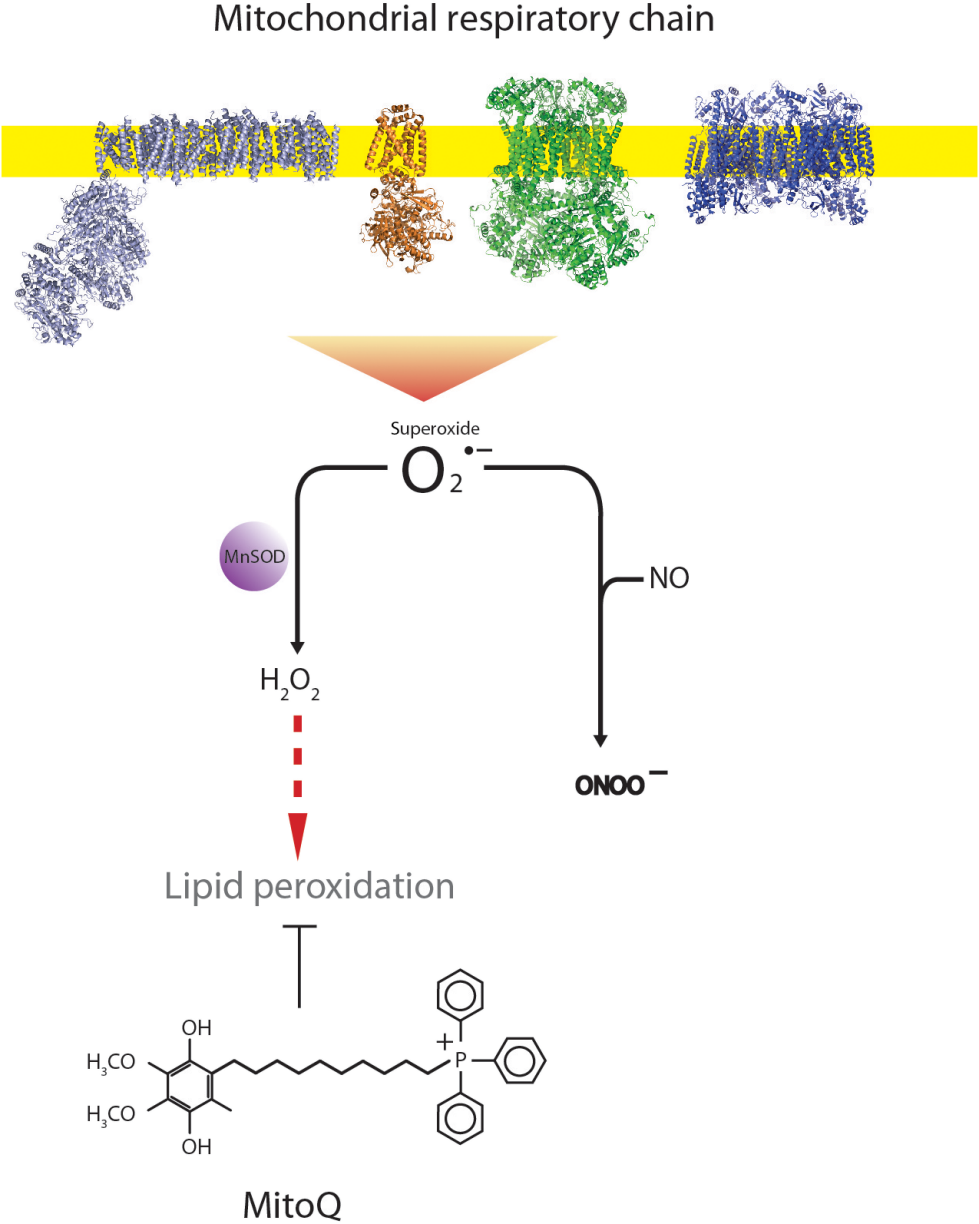
The mitochondria-targeted antioxidant MitoQ prevents lipid peroxidation without affecting superoxide generation. The mitochondrial respiratory chain produces the ROS superoxide (O_2_^•−^), much of which is converted to hydrogen peroxide by the action of MnSOD (Mn superoxide dismutase). The superoxide can also react with NO to form peroxynitrite. The hydrogen peroxide can initiate oxidative damage to the mitochondria in the form of lipid peroxidation. The mitochondria-targeted antioxidant MitoQ blocks lipid peroxidation but does not directly affect mitochondrial superoxide production or react to any substantial extent with superoxide.

Mitochondria are a major site of reactive oxygen species (ROS) production; therefore, protecting them from oxidative damage should be a highly effective therapeutic strategy (*14*, *15*). However, conventional antioxidants are ineffective because they cannot readily penetrate the mitochondria (*14*, *15*). The mitochondria-targeted ubiquinone, MitoQ, overcomes this problem as it is composed of a lipophilic triphenylphosphonium cation covalently attached to a ubiquinol antioxidant (*14*, *15*). Lipophilic cations can easily move through phospholipid bilayers without requiring a specific uptake mechanism. Therefore, the triphenylphosphonium cation concentrates MitoQ several hundred-fold within the mitochondria, driven by the large mitochondrial membrane potential (*14*, *15*). Within mitochondria, superoxide is produced by the respiratory chain and much of the superoxide is converted to hydrogen peroxide, while some of the superoxide also releases ferrous iron from the mitochondrial enzyme aconitase. Together, the hydrogen peroxide and ferrous iron initiate lipid peroxidation in the mitochondrial inner membrane, which contains a large number of unsaturated fatty acids that are particularly susceptible to lipid peroxidation. This lipid peroxidation disrupts and damages mitochondrial function. MitoQ does not affect superoxide production by the respiratory chain. Instead, it is reduced by succinate dehydrogenase to its active ubiquinol form, which is a particularly effective chain-breaking antioxidant that prevents lipid peroxidation and is then continually recycled by succinate dehydrogenase. Thus, MitoQ does not affect superoxide production, but instead, it acts downstream of this superoxide anion production by preventing the damaging lipid peroxidation and mitochondrial damage that is initiated by superoxide (Fig. 1) (*14*, *15*). Therefore, MitoQ may be the candidate antioxidant of choice to protect against programmed cardiovascular disease in offspring of hypoxic pregnancy while maintaining fetal brain sparing. The benefits of MitoQ protecting against pathology have now been revealed in a range of studies in rodents and in two phase 2 human trials (*16*–*20*). However, whether MitoQ treatment in development complicated by chronic hypoxia protects the fetus from oxidative stress and the adult offspring from programmed hypertension in animal models of similar developmental milestones to humans is unknown.

In this study, we have combined experiments in pregnant sheep and in the chicken embryo, both species of comparable temporal cardiovascular development to humans (*11*, *21*), to determine whether MitoQ (i) crosses the placenta and maintains fetal brain sparing, (ii) protects against fetal growth restriction and programmed adult-onset hypertension in the offspring of hypoxic pregnancy, and (iii) directly protects against fetal mitochondria-derived oxidative stress, measured in vivo using a mitochondria-targeted ratiometric mass spectrometry probe (*22*).

## RESULTS

### Maternal MitoQ administration crosses the placenta and does not affect the fetal brain–sparing hemodynamic response in sheep

**Fig. 2:**
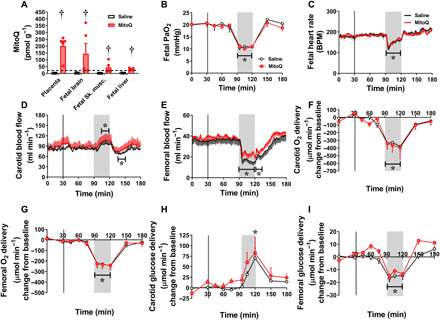
Effect of MitoQ treatment on the brain-sparing response to acute hypoxia in fetal sheep. MitoQ administered intravenously to pregnant ewes at 127 ± 1 days of gestation (term is ca. 145 days) crossed the placenta and accumulated to levels above therapeutic concentrations (dotted line) in the placenta and fetal organs (**A**). During an episode of acute hypoxia [gray box, (**B**)] induced 1 hour after maternal MitoQ treatment (vertical line), fetuses exposed to MitoQ (red group, *n* = 6) compared to saline controls (white group; *n* = 6) showed similar bradycardia (**C**) and equivalent responses in carotid (**D**) and femoral (**E**) blood flow, oxygen (**F** and **G**), and glucose (**H** and **I**) delivery. Data in (C) to (E) represent minute averages with associated SEM throughout the experiment, with overlaid statistical analysis from 15-min epochs. (B) and (F) to (I) represent data taken from a blood sample at each time point (means ± SEM). The effect of MitoQ was determined with a repeated-measures ANOVA with time as a repeated factor and the Tukey’s post hoc test, with the exception of (A), where a two-way ANOVA for treatment and organ was performed. (A) † indicates a significant effect of MitoQ. (B to I) * indicates a significant difference compared to baseline. BPM, beats per minute.

The chronically instrumented pregnant sheep preparation is the animal model system of choice for translational studies on in vivo fetal cardiovascular function of human clinical relevance (*8*, *11*, *12*, *13*). This is because it is the only species in which substantial maternal and fetal surgical instrumentation with catheters and flow probes has been established in late gestation (*8*, *11*). Of added value, the maternal metabolic investment in the pregnancy is similar between sheep and humans (*8*, *11*). We show that a single dose of MitoQ into the maternal venous system at 127 ± 1 days of gestation (term is ca. 145 days) can cross the placenta and accumulate to therapeutic concentrations in fetal tissues (Fig. 2A). We next tested if this therapeutic concentration of MitoQ affected the fetal capacity to respond to an episode of acute stress. Acute hypoxia is a common challenge to the fetus in late gestation, often associated with a mild fetal metabolic acidosis (*8*, *13*). In response to acute hypoxia at 127 ± 1 days of gestation in the chronically instrumented sheep fetus, lowering the fetal PaO_2_ (partial pressure of arterial oxygen) in the descending aorta to 10 to 12 mmHg (Fig. 2B and table S1A), there was a fall in fetal pH and in fetal heart rate, an increase in carotid blood flow, and a fall in femoral blood flow (Fig. 2, C to E). These responses help redistribute blood flow as well as oxygen and glucose delivery away from peripheral circulations toward the fetal brain (Fig. 2, F to I) (*8*). Maternal MitoQ treatment did not affect the fetal blood gas, acid base, or circulatory responses to acute hypoxia (Fig. 2 and table S1A).

### Maternal MitoQ treatment restores fetal growth and protects against a fetal origin of cardiovascular dysfunction in chronically hypoxic sheep

**Fig. 3:**
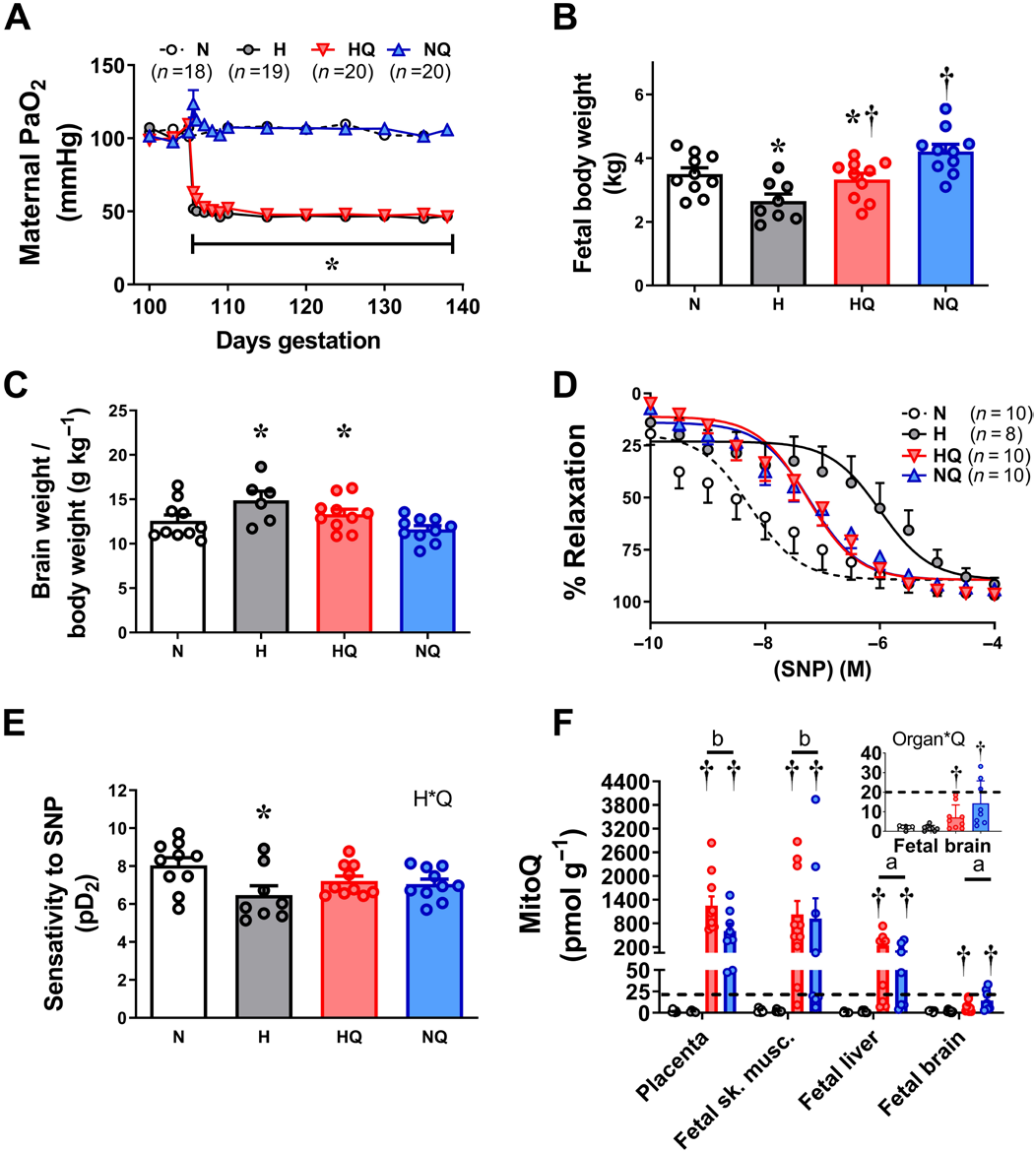
Effect of chronic hypoxia and MitoQ treatment in fetal sheep. (**A**) Exposure to chronic hypoxia from 105 to 138 days of gestation (term is ca. 145 days) decreased the maternal PaO_2_. Fetuses of chronic hypoxic pregnancies (gray) relative to controls (white) had lower fetal body weight (**B**), increased fetal brain weight–to–body weight ratio (**C**), and decreased sensitivity of the fetal femoral artery to the NO donor SNP (**D** and **E**). Maternal daily intravenous treatment with MitoQ to normoxic (blue) or hypoxic (red) pregnant ewes from 105 to 138 days of gestation resulted in greater than therapeutic concentrations (dotted line) of MitoQ content in the placenta and fetal sheep tissues, with the exception of the fetal brain [(**F**), main and magnified inset]. Maternal treatment with MitoQ in hypoxic pregnancy restored fetal body weight (B) while maintaining an elevated fetal brain to fetal body weight ratio (C) and protected against the effects of hypoxia on fetal femoral vascular sensitivity to SNP (D and E). Values are means ± SEM. The effect of hypoxia and MitoQ treatment was determined by two-way ANOVA (B, C, and E), with repeated measures where appropriate (A). * indicates a significant effect of hypoxia; † indicates a significant effect of MitoQ. The effect of MitoQ, hypoxia, and organ type in (F) was determined by three-way ANOVA. Organ*Q indicates a significant interaction, with subsequent post hoc Tukey test. Different letters show significantly different contents of MitoQ by organ type.

Having established that MitoQ administered acutely to the mother can cross the placenta to achieve therapeutic concentrations in the sheep fetus without affecting the fetal circulatory brain–sparing defense response measured in vivo, the next step was to determine if daily maternal MitoQ administration would protect the chronically hypoxic sheep fetus against fetal growth restriction and a fetal origin of cardiovascular dysfunction. While experimental models that restrict uterine blood flow or placental function impair both fetal nutrition and fetal oxygenation in mammals, the isolated effect of chronic hypoxia on the fetus can be best studied by exposing pregnant sheep to an environment of reduced oxygenation. We therefore created four isobaric chambers able to maintain pregnant sheep for long periods of gestation under tightly controlled hypoxic conditions (*12*, *23*, *24*). Exposure of pregnant sheep to a 10% inspired fraction of oxygen for a month, in the last third of gestation, from 105 to 138 days (term is ca. 145 days), led to a controlled reduction in the maternal PaO_2_ and SatHb (hemoglobin O_2_ saturation) (Fig. 3A and table S1B). Chronic hypoxia in untreated and treated ewes led to a transient maternal respiratory alkalosis at 106 days of gestation, the day after the onset of hypoxia (table S1B). Ewes exposed to chronic hypoxia then compensated and showed a significant increase from baseline in maternal [Hb], irrespective of treatment (table S1B). Chronic hypoxia in pregnant sheep led to fetal growth restriction with evidence of fetal brain sparing, as demonstrated by an increase in the fetal brain weight relative to body weight (Fig. 3, B and C, and table S2). In contrast to the fetal brain, the fetal heart and the fetal liver weight–to–body weight ratio were not altered by chronic hypoxia (table S2). The dilator response of isolated third-order femoral arterial segments to the NO donor sodium nitroprusside (SNP) was diminished in the chronically hypoxic sheep fetus (Fig. 3, D and E). Maternal daily MitoQ treatment, from 105 to 138 days of gestation, resulted in greater accumulative therapeutic concentrations of MitoQ in the placenta, fetal skeletal muscle, and fetal liver than with single administration. In contrast, the concentration of MitoQ in the fetal brain was lower when measured later compared to earlier in gestation (Fig. 3F). Maternal chronic treatment with MitoQ in hypoxic pregnancy restored fetal growth while maintaining fetal brain sparing and improved the fetal femoral NO-dependent dilator response (Fig. 3, B to E, and table S2). Maternal chronic MitoQ treatment in normoxic pregnancy did not have any effect on fetal cardiovascular function (Fig. 3, B to E) or biometry, with the exception of a small reduction in the fetal brain weight (−3.6%) and increasing the fetal liver weight (table S2).

### MitoQ treatment prevents in vivo mitochondria-derived oxidative stress and protects against cardiovascular dysfunction in the chronically hypoxic chicken embryo

**Fig. 4:**
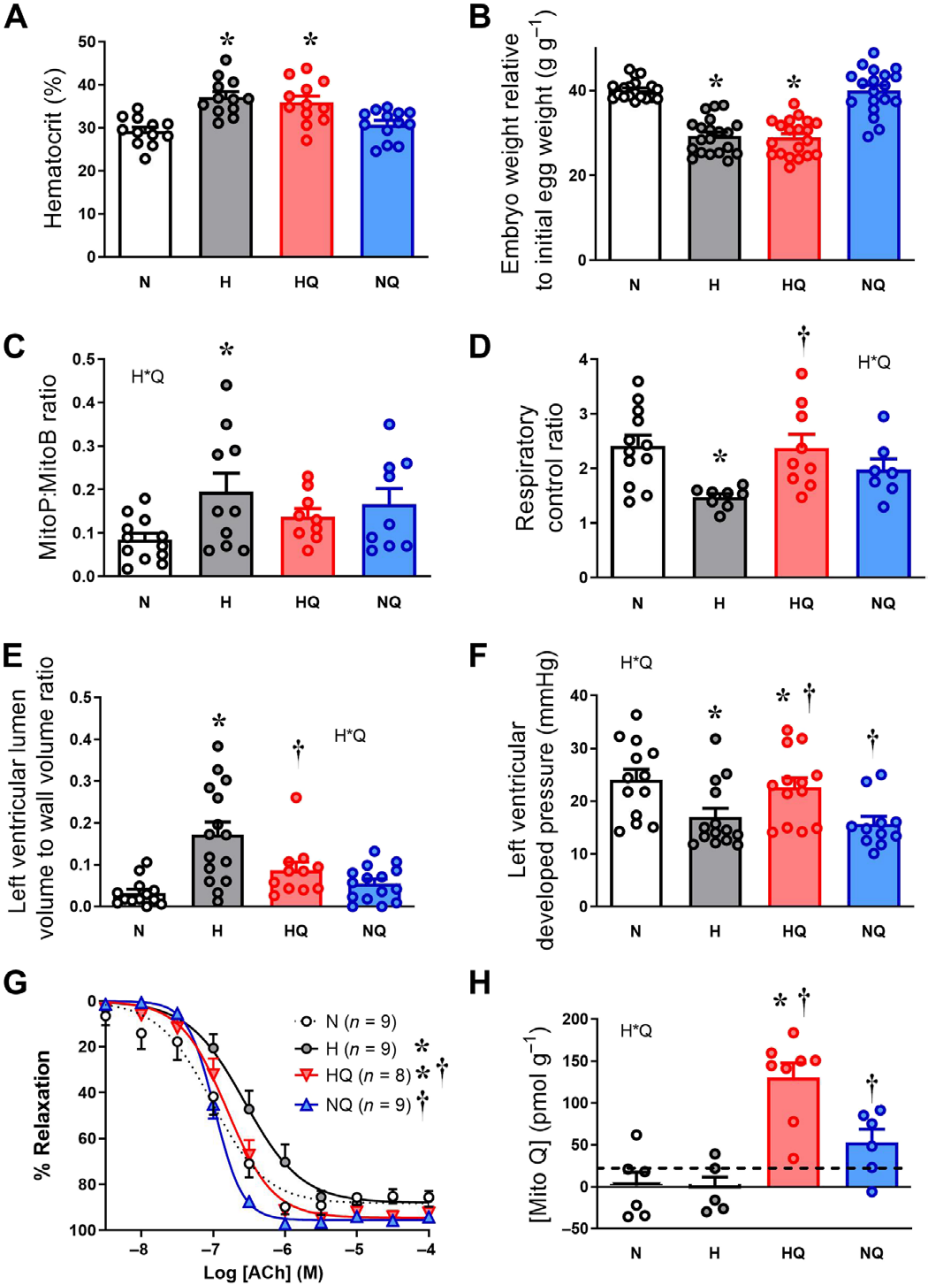
Effect of chronic hypoxia and MitoQ treatment in the chicken embryo. Compared to normoxic embryos (white), incubation of chicken embryos under hypoxia (gray) from 0 to 19 days (term is 21 days) increased hematocrit (**A**) and caused growth restriction (**B**). Chronic hypoxia increased mitochondria-derived oxidative stress measured in vivo (conversion of MitoP to MitoB) in the chicken embryo heart (**C**), decreased the cardiac mitochondrial respiratory control ratio (**D**), increased the left ventricular lumen to wall ratio (**E**), reduced the LVDP in an isolated Langendorff preparation (**F**), and impaired endothelial dependent vasorelaxation to ACh in isolated femoral arteries via in vitro wire myography (**G**). Treating chicken embryos with MitoQ between days 13 and 18 of incubation resulted in a significant elevation of MitoQ content above therapeutic levels (dotted line) in the fetal heart in both hypoxic (red) and normoxic (blue) groups (**H**). MitoQ treatment in hypoxic chicken embryos did not affect the elevation in hematocrit or the growth restriction, but it restored all other outcomes (A to G). Values are means ± SEM. The effect of hypoxia and MitoQ treatment was determined by two-way ANOVA, with repeated measures where appropriate (G). * indicates a significant effect of hypoxia; † indicates a significant effect of MitoQ; H*Q indicates a significant interaction, with subsequent post hoc Tukey test. Statistics for (G) reflect comparisons of area above the curve.

To isolate direct effects of MitoQ on the developing offspring under hypoxic conditions, we used the chicken embryo, the only established animal model system in which the direct effects of treatment on the fetal heart and circulation can be established, independent of effects on the mother and/or the placenta (*21*). Of added value, chickens and humans also share comparable developmental milestones in cardiovascular physiology (*21*). We found that incubation of fertilized chicken eggs under chronic hypoxia (14 ± 0.5% O_2_) from the start of incubation until day 19 (term is 21 days) increased the percentage of hematocrit in blood (Fig. 4A) and reduced body weight (Fig. 4B). We determined mitochondria-derived oxidative stress within the living organism using a mitochondria-targeted ratiometric mass spectrometry probe (*22*). We found that hypoxic incubation increased the MitoP:MitoB ratio in the chicken embryo heart, indicating enhanced in vivo mitochondria-derived oxidative stress during hypoxic development (Fig. 4C). Measurement of mitochondrial respiration in saponin-permeabilized isolated heart muscle fibers at day 19 of incubation revealed a decrease in the mitochondrial respiratory control ratio in hypoxic chicken embryos (Fig. 4D). Stereological analysis of perfusion-fixed hearts and functional analysis of freshly isolated hearts and femoral vessels in different cohorts of chicken embryos also revealed changes in the structure and function of the cardiovascular system. Chicken embryos incubated under hypoxic conditions showed an increase in the left ventricular lumen to wall volume ratio (Fig. 4E), with evidence of systolic dysfunction (Fig. 4F), and a rightward shift in the femoral vasodilator response to acetylcholine (ACh), confirming endothelial dysfunction (Fig. 4G). Administration in ovo of MitoQ onto the chorioallantoic membrane resulted in elevated concentrations of MitoQ in hearts of both normoxic and hypoxic chicken embryos (Fig. 4H). MitoQ treatment of hypoxic chicken embryos did not affect the increase in hematocrit or the reduction in body weight (Fig. 4, A and B). However, it prevented the enhanced in vivo mitochondria-derived oxidative stress in the embryonic heart and restored the cardiac mitochondrial respiratory control ratio, left ventricular structure, and systolic function (Fig. 4, C to F). MitoQ treatment in the hypoxic chicken embryo also improved endothelial function (Fig. 4G), an effect that could not be attributed to the MitoQ carrier (fig. S1). MitoQ treatment of normoxic chicken embryos did not have any effect on embryonic weight or on outcome variables relating to embryonic cardiovascular structure and function, with the exception of decreasing left ventricular developed pressure (LVDP) (Fig. 4F).

### Maternal MitoQ treatment during hypoxic pregnancy in sheep protects against programmed hypertension in the adult offspring by enhancing in vivo NO signaling

**Fig. 5:**
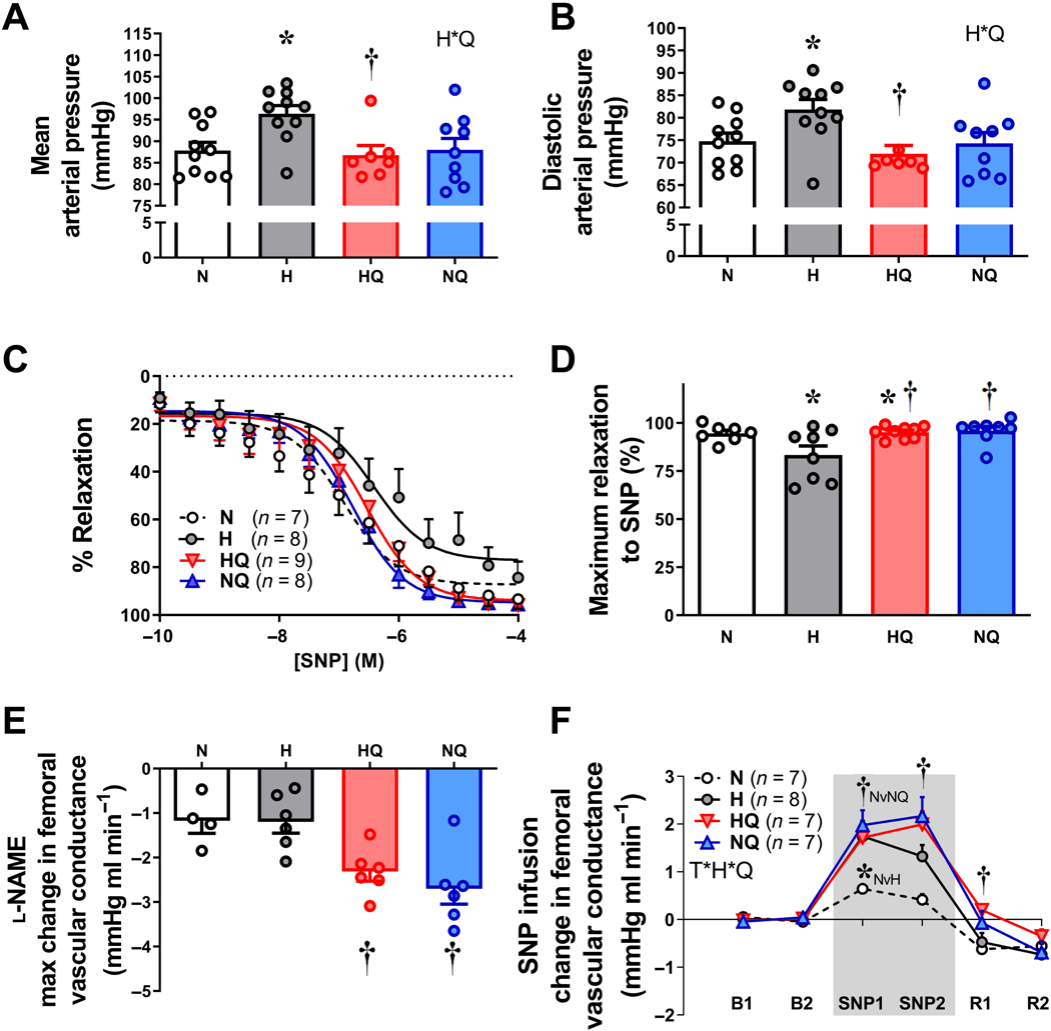
Effect of chronic hypoxia and MitoQ treatment on the programming of vascular dysfunction and hypertension in the adult offspring in sheep. Compared to controls (white), young adult lambs of hypoxic pregnancy (gray) were hypertensive (**A** and **B**) by 9 months. (**C** and **D**) Their isolated femoral arteries showed impaired relaxation to SNP (wire myography). Maternal MitoQ treatment in hypoxic pregnancy (red) restored the programmed hypertension (A and B) and improved the SNP femoral relaxation (C and D). Maternal MitoQ in normoxic pregnancy (blue) did not affect arterial pressure or SNP relaxation (A to D). Maternal MitoQ treatment in any pregnancy programmed an increase in basal NO bioavailability in the adult offspring measured in vivo. (**E**) A greater fall in FVC in response to l-NAME treatment supports this. Maternal MitoQ treatment in any pregnancy also programmed an increase in peripheral vascular sensitivity to NO in the adult offspring in vivo. (**F**) A greater increment in FVC in response to SNP infusion (gray area) for 30 min supports this. Values are means ± SEM. The effect of hypoxia and MitoQ treatment was determined by two-way ANOVA, with repeated measures where appropriate (C and F). * indicates a significant effect of hypoxia; † indicates a significant effect of MitoQ. T*H*Q indicates significant interaction, with subsequent post hoc Tukey or Students *t* test. For (F), the average of 15-min epochs is presented for baseline (B1 and B2), SNP (SNP1 and SNP2), and recovery (R1 and R2).

To then determine the effect of MitoQ treatment on long-term cardiovascular health in adult offspring, a second cohort of pregnant ewes undergoing control or chronic hypoxic pregnancy were allowed to give birth naturally. Ewes and their lambs from treated and untreated normoxic or hypoxic pregnancies were put out to pasture, and lambs were maintained until 9 months of age under normoxic conditions at the Barcroft Centre (Cambridge, UK, sea level). At 9 months of age, we established the cardiovascular health of the young adult offspring via surgically implanted vascular catheters and a femoral flow probe in chronically stable preparations. We found that adult offspring of ovine hypoxic pregnancy had systemic hypertension (Fig. 5, A and B). The impaired femoral dilator response to NO discovered in fetal life was still evident in adult lambs of hypoxic pregnancy (Fig. 5, C and D). Maternal treatment with MitoQ in ovine hypoxic pregnancy prevented the programmed systemic hypertension in the adult offspring (Fig. 5, A and B). We determined whether the mechanism underlying MitoQ protection against programmed hypertension in adult lambs of hypoxic pregnancy was by enhancing NO pathways. MitoQ treatment in ovine hypoxic pregnancy restored the programmed NO-dependent dilatation in femoral arterial segments isolated from adult lambs (Fig. 5, C and D). In addition, in vivo treatment of adult lambs with a bolus dose of the NO synthase blocker N^G^-nitro-l-arginine methyl ester (l-NAME) led to a greater fall in femoral vascular conductance (FVC) in adult offspring of MitoQ-treated pregnancies, irrespective of oxygenation (Fig. 5E). Last, in vivo intravenous infusion with the NO donor SNP promoted a greater increase in FVC in adult lambs of MitoQ-treated pregnancies, also irrespective of oxygenation (Fig. 5F). Adult offspring of MitoQ-treated normoxic ovine pregnancy no longer showed effects on brain or liver weight found in fetal life (table S2).

## DISCUSSION

**Fig. 6:**
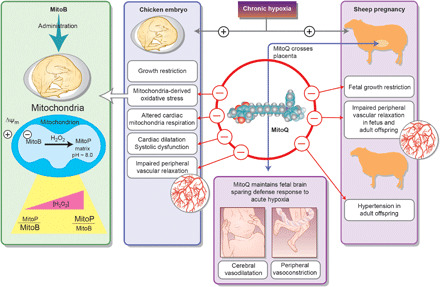
Summary illustration: Protective effects of MitoQ treatment in hypoxic development. The mitochondria-targeted antioxidant MitoQ protects the chronically hypoxic fetus from cardiovascular dysfunction and remodeling, which programs adult-onset peripheral vascular dysfunction and systemic hypertension. These protective effects of MitoQ in the compromised fetus occur without affecting the fetal brain–sparing hemodynamic response to acute hypoxia. Mechanisms underlying benefits of MitoQ on the offspring cardiovascular system include direct protection against mitochondria-derived oxidative stress and normalization of cardiac mitochondrial respiration in the hypoxic embryo, together with programmed increases in circulating NO bioavailability and in peripheral vascular sensitivity to it in the adult offspring. MitoQ treatment in sheep protects against fetal growth restriction in hypoxic pregnancy. In contrast, MitoQ treatment in hypoxic chicken embryos does not affect growth restriction. Therefore, protective effects of MitoQ on fetal growth in mammals may occur at the level of the placenta.

In this study, by addressing questions at the in vivo, isolated organ, cellular, mitochondrial, and molecular levels and exploiting the strength of two animal models, we provide a comprehensive approach to understanding the mechanisms linking MitoQ protection against fetal growth restriction and programmed cardiovascular dysfunction in offspring following development complicated by chronic hypoxia. The data show that MitoQ can protect the chronically hypoxic fetus from fetal growth restriction, fetal cardiac and peripheral vascular dysfunction, and programmed adult-onset systemic hypertension. These protective effects of MitoQ on the compromised offspring occur without affecting a major fetal defense mechanism during acute stress, the fetal brain–sparing hemodynamic response. Mechanisms underlying benefits of MitoQ on the offspring of hypoxic pregnancy include direct protection against mitochondria-derived oxidative stress in vivo, normalization of cardiac mitochondrial respiration, and programmed increases in NO signaling (Fig. 6).

Episodes of oxygen deprivation in the mature mammalian fetus are common, resulting from transient compression of the umbilical cord in late gestation or during uterine contractions in labor and delivery (*8*). The fetal defense response to acute hypoxia is triggered by a fetal carotid chemoreflex (*25*) and involves fetal bradycardia, an increase in fetal carotid blood flow, and a fall in fetal femoral blood flow (*8*, *25*). The fetal bradycardia reduces myocardial oxygen consumption (*8*, *26*), and the redistribution of the fetal cardiac output prioritizes blood flow, oxygen, and glucose delivery to the fetal brain at the expense of the peripheral circulation—the so-called fetal brain–sparing effect (*8*, *25*). In contrast to the fetal cerebral circulation, in which the vasodilator effects of hypoxia are mediated via local increases in dilator agonists, the mechanisms mediating fetal peripheral vasoconstriction during hypoxia involve neural, endocrine, and local redox responses (*8*). Peripheral vasoconstriction in the fetus is triggered by a carotid chemoreflex (*25*) and is maintained via the release of constrictor hormones into the fetal circulation (*8*). In addition, we have shown that a local redox response to hypoxia in the fetal peripheral vasculature is created by an elevated ratio of O_2_^•−^ to NO (*8*, *13*). Consequently, fetal treatment with conventional antioxidants, such as vitamin C (*13*), allopurinol (*27*), or melatonin (*28*), or with other agents that quench O_2_^•−^ and/or increase NO bioavailability in the fetal circulation, such as statins (*29*), all reduce the ratio of O_2_^•−^ to NO and weaken the fetal femoral vasoconstrictor response to acute hypoxia. In contrast, here, we show that maternal treatment with MitoQ is different, as it maintains an intact fetal peripheral vasoconstrictor response to acute hypoxia. Therefore, the data support that MitoQ treatment in hypoxic pregnancy does not affect superoxide production. Instead, it acts downstream of this superoxide anion production by preventing the damaging lipid peroxidation and mitochondrial damage that is initiated by superoxide (Fig. 1) (*14*, *15*). On one hand, the maintenance of superoxide anion production during hypoxic conditions in fetal life by MitoQ permits O_2_^•−^ to react with NO in the fetal peripheral vasculature. This maintains the contribution of the local redox response to the fetal peripheral vasoconstriction, part of the fetal brain–sparing effect. On the other hand, prevention by MitoQ of the damaging lipid peroxidation and mitochondrial damage that is initiated by superoxide protects against the developmental programming of cardiovascular disease in the adult offspring of hypoxic pregnancy. Therefore, the combined effects of MitoQ treatment in hypoxic pregnancy make it ideal to maintain beneficial physiological roles of ROS generation in the fetal circulation while protecting from the programming effects of oxidative stress on the developing cardiovascular system.

One of the most common outcomes of pregnancy complicated by chronic fetal hypoxia in humans is fetal growth restriction (*5*, *8*). In humans, low birth weight is related not only to poor neonatal outcome (*30*) but also with endothelial dysfunction (*31*). In turn, peripheral vascular dysfunction is a harbinger of systemic hypertension and increased susceptibility to cardiovascular disease (*32*). To date, there is no cure for human adverse pregnancy associated with fetal growth restriction or programmed cardiovascular dysfunction in the adult offspring. We show that MitoQ crosses the placenta in ovine pregnancy to achieve therapeutic concentrations in the sheep fetus and that maternal treatment with MitoQ in hypoxic pregnancy protects against fetal growth restriction, as well as programmed peripheral vascular dysfunction and hypertension in the adult offspring. We adopted a three-pronged approach to address the mechanisms underlying the protection conferred by MitoQ on programmed cardiovascular dysfunction in offspring of hypoxic pregnancy. First, experiments using in vitro wire myography show that maternal treatment with MitoQ can restore the programmed impairment in NO sensitivity in femoral vessels isolated from adult offspring of hypoxic pregnancy (Fig. 5, C and D). Second, experiments in vivo with l-NAME treatment show that maternal treatment with MitoQ during pregnancy can program a greater contribution of NO to basal femoral vascular tone in the circulation of adult offspring (Fig. 5E). Third, experiments in vivo with SNP treatment show that maternal treatment with MitoQ can program a greater femoral dilator response in adult offspring (Fig. 5F). Combined, these data support that programmed increases in both NO bioavailability and NO sensitivity provide a molecular link explaining the protective effects of maternal treatment with MitoQ against hypertension in the adult offspring of hypoxic pregnancy in sheep. Of interest, the concentration of MitoQ measured within the fetal brain following treatment at 127 ± 1 days of gestation was lower than that measured at 138 days of gestation (term is ca. 145 days). A likely explanation is the developmental increase in the abundance and activity of the efflux transporter p-glycoprotein on the blood brain barrier toward term. Previous studies in adult rodents have demonstrated that MitoQ concentrations in the brain are lower than other tissues (*14*, *15*).

The mitochondria-targeted ratiometric mass spectrometry probe MitoB can measure products of mitochondrial oxidative stress in living cells (*22*). The probe, MitoB, comprises a triphenyl phosphonium (TPP) cation driving its accumulation within mitochondria, conjugated to an arylboronic acid that reacts with H_2_O_2_ to form a phenol, MitoP. Therefore, quantifying the MitoP:MitoB ratio enables measurement of oxidative stress in vivo derived from the mitochondria within cells of tissues at the time of MitoB injection (*22*). In the present experiments in the chicken embryo, the MitoP:MitoB ratio predominantly reported retrospectively on in vivo cardiac mitochondria–derived oxidative stress in the living hypoxic embryo. Not only these findings isolate direct adverse effects of chronic hypoxia on fetal cardiac and vascular function, independent of maternal and/or placental exposure to reduced oxygenation, but also the data demonstrate that we can report retrospectively in vivo mitochondria-derived oxidative stress in the living hypoxic fetus. Because direct MitoQ treatment of the chicken embryo did not prevent growth restriction during hypoxic incubation, but maternal MitoQ treatment in sheep restored fetal growth in hypoxic pregnancy, the protective effects of MitoQ specifically on fetal growth in mammals appear to be at the level of the placenta. Accordingly, we and others have reported in a rat model that MitoQ treatment in hypoxic pregnancy prevents markers of oxidative stress and of activation of the unfolded protein response pathways in the placenta (*18*, *20*, *33*). MitoQ treatment in hypoxic pregnancy also increased placental maternal blood space surface area and volume (*33*). Therefore, MitoQ treatment in hypoxic pregnancy protects against placental stress and enhances placental perfusion, maintaining oxygen and nutrient delivery for fetal growth (*18*, *20*, *33*). Alternatively, the protective effects of MitoQ on fetal growth in ovine but not avian hypoxic development may reflect placental independent effects. An example may be possible differences in the direct effects of mitochondria-targeted antioxidants on fetal growth between mammals and birds. However, we could not find any supporting evidence.

In conclusion, our discoveries provide compelling evidence of clinical translational importance for early development compromised by chronic fetal hypoxia and the programming of hypertension in the offspring in species of similar developmental milestones to humans. We provide insight into underlying mechanisms and thereby successful intervention against cardiovascular dysfunction in the next generation programmed by hypoxic pregnancy. The mitochondria-targeted antioxidant intervention prevents programmed cardiovascular dysfunction in the offspring while maintaining the fetal brain–sparing effect. Therefore, MitoQ is a plausible candidate for antioxidant therapy of improved human translational capacity that protects offspring of high-risk pregnancy from programmed cardiovascular dysfunction in later life.

## MATERIALS AND METHODS

All procedures were performed under the Home Office Project Licence PL70/7645 and PL80/2232 (sheep) and P0592D78B (chickens) under the UK Animals (Scientific Procedures) Act 1986, following critical ethical review by the University of Cambridge Animal Welfare and Ethical Review Board.

### Experiments in sheep

#### General surgical preparation

Food, but not water, was withdrawn for 24 hours before surgery. General anesthesia was induced in pregnant ewes and young adult offspring (9-month-old lambs) with alfaxalone [2 to 3 mg kg^−1^, intravenously (iv); Alfaxan, Jurox] and maintained with isoflurane (1.5 to 2.0% in 4:1 O_2_:N_2_O) using a positive pressure ventilator (Datex-Ohmeda Ltd., Hatfield, Hertfordshire, UK) (*14*, *24*, *34*). Oxygen saturation, end-tidal carbon dioxide, and heart rate were noninvasively monitored throughout. Antibiotics [30 mg kg^−1^ procaine benzylpenicillin, intramuscularly (im); Depocillin; Intervet UK Ltd., Milton Keynes, UK], analgesic (1.4 mg kg^−1^ carprofen, sc; Rimadyl; Pfizer Ltd., Kent, UK), and vitamin B12 (0.04 mg kg^−1^ Anivit B12, im; Animalcare Ltd., York, UK) were administered immediately before the start of surgery.

#### Surgical preparations for acute hypoxia experiments

Six Welsh Mountain ewes carrying a singleton pregnancy determined by ultrasound scan (Toshiba Medical Systems Europe, Zoetermeer, The Netherlands) were surgically instrumented for the purpose of long-term recording at 117 ± 2 days of gestational age (term is ca. 145 days), as previously described in detail (*12*, *13*, *24*, *27*–*29*, *34*). Midline abdominal and uterine incisions were made to exteriorize either the fetal hind limbs or head. Fetal femoral and carotid arterial catheters [inside diameter (ID), 0.86 mm; outer diameter (OD), 1.52 mm; Critchley Electrical Products, Kingsgrove, NSW, Australia] were inserted. Another catheter was anchored onto the fetal hind limb for recording of the reference amniotic pressure. Transonic flow transducers were positioned around the contralateral femoral and carotid artery (2-mm aperture; R-series, Transonic Systems Inc.) (*25*). The uterine and abdominal incisions were closed in layers, following which maternal femoral arterial (Teflon; ID, 1.0 mm; OD, 1.6 mm; Altec, UK) and venous [polyvinyl chloride (PVC); ID, 0.86 mm; OD, 1.52 mm; Critchley Electrical Products, NSW, Australia] catheters were inserted. All catheters and flow transducers were exteriorized through a keyhole incision on the flank to be kept inside a plastic pouch sewn onto the skin. The dead space of all catheters was filled with heparinized saline (100 IU/ml heparin in 0.9% NaCl), and the catheter ends were closed with three-way taps. Inhalation anesthesia was withdrawn, and the ewe was extubated only after spontaneous breathing had returned (*12*, *13*, *24*, *27*–*29*, *34*).

#### Surgical preparation for chronic hypoxia experiments

Seventy-four Welsh Mountain ewes carrying a singleton pregnancy were surgically instrumented at 100 ± 1 days of gestational age. Following a midline abdominal incision and uterotomy, the fetal hind limbs were exposed and the fetal sex was determined, as before (*12*). To make the study viable ethically and economically, every singleton ovine pregnancy generated was used. Therefore, studies in the fetal period used the male offspring (*n* = 38), while studies in the adult period used the female offspring (*n* = 36), as ewe lambs are easier to group house and maintain compared to rams. Longitudinal comparisons from the fetal to the adult period therefore require caution. The male or female fetus was returned into the uterine cavity, and the uterine and maternal abdominal incisions were closed in layers. Following which, maternal femoral arterial (Teflon; ID, 1.0 mm; OD, 1.6 mm; Altec, UK) and venous (PVC; ID, 0.86 mm; OD, 1.52 mm; Critchley Electrical Products, NSW, Australia) catheters were inserted (*12*, *13*, *24*, *27*–*29*, *34*).

#### Surgical preparation of adult offspring

Young adult lambs (*n* = 36) were surgically instrumented at 9 months of age, as previously described in detail (*12*). In brief, femoral arterial (Teflon; ID, 1.0 mm; OD, 1.6 mm; Altec, UK) and venous (PVC; ID, 0.86 mm; OD, 1.52 mm; Critchley Electrical Products, NSW, Australia) catheters were inserted and Transonic flow transducers were placed around the contralateral femoral artery (4 mm; SB-series, Transonic Systems Inc.). All catheters and flow transducers were exteriorized through a keyhole incision on the flank to be kept inside a plastic pouch sewn onto the skin. The dead space of all catheters was filled with heparinized saline (100 IU/ml heparin in 0.9% NaCl), and the catheter ends were closed with three-way taps. Inhalation anesthesia was withdrawn, and the lamb was extubated only after spontaneous breathing had returned (*12*).

#### Postoperative care

During 5 days of postsurgical recovery, pregnant ewes and young adult lambs were housed in individual floor pens with a 12-hour:12-hour light:dark cycle, hay, and water ad libitum and were fed concentrates twice daily (100 g of sheep nuts no. 6; H & C Beart Ltd., Kings Lynn, UK). Antibiotics were administered daily for 5 days after surgery to the ewe and adult offspring (30 mg kg^−1^ procaine benzylpenicillin, im; Depocillin; Intervet UK Ltd.) and daily to acute hypoxia fetuses into the amniotic cavity (600 mg in 2 ml of 0.9% NaCl, benzylpenicillin; Crystapen, Schering-Plough, Animal Health Division, Welwyn Garden City, UK). Analgesia was administered to pregnant ewes and young adult lambs for 3 days after surgery [1.4 mg kg^−1^ carprofen, subcutaneously (sc); Rimadyl; Pfizer Ltd.]. Daily arterial blood samples were taken from the pregnant ewe, fetus, and young adult lambs to monitor well-being. The blood samples were analyzed immediately for pH and partial pressures of O_2_ and CO_2_, corrected for maternal/young adult lamb (38°C) or fetal (39.5°C) body temperature, using an ABL5 radiometer (Radiometer Copenhagen, Crawley, UK) (*12*, *13*, *24*, *27*–*29*, *34*). Hb, SatHb, and percentage hematocrit were measured using an ABL80 hemoximeter (Radiometer Copenhagen, Crawley, UK). Blood glucose concentration was measured on a YSI blood analyzer (YSI 2300 STAT Plus, Yellow Springs Instruments, OH, USA), and all catheters were flushed with heparinized saline (100 IU/ml heparin in 0.9% NaCl).

#### In vivo materno-fetal transfer of MitoQ and acute hypoxia

Following 4 days of postoperative recovery, ewes were transferred to metabolic crates. Fetal and maternal arterial catheters were connected to sterile pressure transducers (COBE; Argon Division, Maxim Medical, Athens, TX, USA) and Transonic flow probes connected to a flow meter (T206; Transonics Systems Inc., Ithaca, NY, USA). Calibrated mean fetal arterial blood pressure (corrected for amniotic pressure), maternal mean arterial blood pressure, fetal and maternal heart rate (triggered via a tachometer from the pulsatility in either the arterial blood pressure or blood flow signals), and mean fetal carotid and fetal femoral blood flow were recorded continually at 1-s intervals using a computerized Data Acquisition System (DAS; Department of Physiology, Cambridge University, UK). After 24 hours of acclimatization, on the fifth and sixth post-operative days, at 127 ± 1 days, all fetuses were subjected to two acute hypoxia experiments, carried out on consecutive days. Each protocol consisted of a 3-hour period divided into 1.5-hour normoxia, 0.5-hour hypoxia, and 1-hour recovery, as previously described (*13*, *27*–*29*, *34*). At 30 min of normoxia (1 hour before hypoxia), ewes were administered either 20 ml of saline (first day) or MitoQ (second day) through their femoral vein catheter over 5 min. To achieve a therapeutic concentration of MitoQ, MS010 (6 mg kg^−1^) was dissolved in saline. This concentration equated to approximately 1.2 mg kg^−1^ of bioactive MitoQ and was based on the largest dose administered in human clinical trials (80 mg kg^−1^ day^−1^; assuming body weight of 67 kg) (*16*). Maternal and fetal arterial blood samples were collected before, during, and after hypoxia as well as before and after infusion to measure blood gases, pH, SatHb, glucose, and Hb. Oxygen content was calculated by the equation (O_2_ saturation/100) × ([Hb] × 1.39) + (PaO_2_ × 0.003) (*35*). Oxygen and glucose delivery were determined by multiplying content/concentration by arterial blood flow. At precisely 1 hour after the MitoQ acute hypoxia protocol, ewes were euthanized with a lethal overdose of anesthetic (200 ml kg^−1^ sodium pentobarbital, iv; Pentoject; Animalcare Ltd., York, UK). The positions of implanted catheters and flow probe were confirmed, and maternal, placental, and fetal tissue biopsies were collected and snap-frozen for subsequent quantification of MitoQ uptake by liquid chromatography–tandem mass spectrometry (LC-MS/MS) (*22*).

#### Chronic hypoxia ± MitoQ

From 103 days of gestation, ewes were fed daily a bespoke maintenance diet made up of concentrate and hay pellets to facilitate the monitoring of food intake (Cambridge ewe diet: 40 g kg^−1^ nuts and 3 g kg^−1^ hay; Manor Farm Feeds Ltd.; Oakham, Leicestershire, UK). On 104 days of gestation, ewes were randomly assigned to either chronic normoxia or hypoxia. If assigned to chronic hypoxia, ewes were transferred to one of four bespoke isobaric chambers (Telstar Ace, Dewsbury, West Yorkshire, UK) under normoxia to acclimatize for 24 hours (*12*, *23*, *24*). If assigned to the normoxic protocol, ewes remained in individual floor pens of the same floor area as the hypoxic chambers. At 105 days of gestation, chronic normoxia or chronic hypoxia was started and continued until 138 days of gestation. The protocol consisted of either normoxia (N) or hypoxia (H; ~10% maternal inspired O_2_, tailored to each ewe to maintain the maternal PaO_2_ between 45 and 55 mmHg) as before (*12*, *23*, *24*). This model of hypoxic pregnancy in sheep does not affect maternal food intake (*12*, *23*). In treated groups, a daily 5-ml bolus of either saline or MitoQ (Q; 6 mg kg^−1^ of MS010 dissolved in saline) was administered. This was the same amount of MitoQ as was given in the acute hypoxia protocol, except administered in this protocol every day for 33 days through the indwelling maternal femoral vein catheter (N: *n* = 20, H: *n* = 18, HQ: *n* = 17, NQ: *n* = 19). Ewes in the fetal cohort were euthanized at 138 ± 1 days of gestation under their respective oxygen conditions 24 hours after the last dose of saline or MitoQ treatment. Ewes in the postnatal cohort received their last dose of MitoQ at 138 days of gestation. If an ewe in the postnatal cohort was hypoxic, the ewe was slowly returned to normoxia precisely 30 min after her last administration of MitoQ at 138 days of gestation. All ewes in the postnatal cohort were allowed to give birth spontaneously under normoxic conditions with food and water ad libitum. Lambs were maintained with their mothers under normoxia, and they are weaned at 5 months of age (*12*). Weaned lambs were maintained under normoxia until 9 months of age.

#### Cardiovascular function in adult offspring

At 272 ± 5 days (9 months) of age, when sheep are sexually mature and classified as young adults (*12*), basal and stimulated cardiovascular function was determined following 5 days of postoperative recovery. Briefly, surgically prepared young adult lambs were moved into a metabolic crate in the morning; their arterial catheter was connected to a sterile pressure transducer (Argon Division, Maxxim Medical, Athens, TX, USA) and their femoral flow probe was connected to a Transonic flow meter (T206; Transonics Systems Inc., Ithaca, NY, USA). Adult offspring were acclimatized to being in the crate for 2 to 3 hours before commencing recordings at a similar time of day. A custom-built DAS (Maastricht–Programmable AcQuisition system, M-PAQ and IDEEQ software, Maastricht Instruments, The Netherlands; 1000-Hz sample rate) was used to record arterial blood pressure. Blood flow signals from the Transonic flow meter were also directly recorded by the IDEEQ software, and heart rate was calculated continuously on-line by the program using the femoral blood flow or systolic pulse as a trigger. Final analysis of IDEEQ files was performed in LabChart (LabChart 7 Pro, AD Instruments Ltd., Chalgrove, UK). Femoral vascular resistance (FVR) and FVC were calculated by applying Ohm’s law to the circulation, using the following equations, where MAP is the mean arterial blood pressure and FBF is femoral blood flow: FVR = MAP/FBF and FVC = FBF/MAP (*12*, *13*, *23*, *24*, *25*, *27*–*29*, *34*).

#### In vivo analysis of peripheral vascular sensitivity to NO in adult offspring

At 9 months of age, the NO donor SNP (Sigma Chemicals; dissolved in heparinized saline) was infused into the femoral vein catheter in adult offspring at a concentration of 2.5 mg kg^−1^ min^−1^ for 30 min. Cardiovascular data were collected as 1-min averages for the 30 min before, during, and after SNP infusion. In particular, we focused on the FVC response (femoral blood flow/arterial blood pressure) to SNP infusion as an in vivo biomarker of the peripheral circulation sensitivity to NO.

#### In vivo analysis of circulating NO bioavailability in adult offspring

At 9 months of age, following at least 2 days after treatment with SNP, a bolus of the NO synthase inhibitor l-NAME (100 mg kg^−1^; Sigma Chemicals; dissolved in 10 ml of heparinized saline) was infused slowly over 30 s into the indwelling femoral artery catheter of the adult offspring. Cardiovascular data were recorded for 15 min before l-NAME treatment and until peak responses in arterial blood pressure and in femoral blood flow were observed. The fall from baseline in FVC (femoral blood flow/mean arterial blood pressure) represented an in vivo biomarker of the circulating NO bioavailability.

#### Postmortem procedures and tissue collection

Pregnant ewes at 138 ± 1 days of gestational age and adult lambs (>5 days after l-NAME treatment) were given a lethal overdose of anesthetic (200 ml kg^−1^ sodium pentobarbital, iv; Pentoject; Animalcare Ltd., York, UK). Body and organ weights were taken from fetal and adult offspring (see table S2). The third-order femoral artery of both fetal and young adult lambs was isolated to assess vascular relaxation ex vivo via wire myography.

#### Wire myography

The third-order femoral artery of fetal and adult lambs was dissected and mounted on a wire myograph (Multi Wire Myograph System 610M; DMT Aarhus, Denmark) (*12*). Data were recorded using PowerLab and LabChart software (LabChart Pro 7.0, PowerLab 8/30; AD Instruments, Chalgrove, UK), and vessels were normalized using the DMT normalization module to 5.30 and 10.66 kPa for fetal sheep and young adult lambs, respectively. In both the ovine fetus and young adult lamb, the vascular responsiveness to SNP (10^−10^ to 10^−4^ M) was assessed following preconstriction with a submaximal dose of norepinephrine.

#### Measurement of MitoQ content in tissue with mass spectrometry

For both the acute hypoxia and chronic hypoxia (fetal cohort) experiments, placentomes, fetal mid-brain, fetal biceps femoris, and fetal liver were snap-frozen in liquid nitrogen at postmortem and stored at −80°C until analysis for levels of MitoQ using LC-MS/MS (*22*). Frozen tissues were homogenized in 50 mM tris buffer (pH 7) and extracted with acetonitrile and evaporation. The extracts were reconstituted, and the MitoQ contents of the tissue were measured using LC-MS/MS. An I-Class Acquity UPLC attached to a Xevo TQ-S triple quadruple mass spectrometer (both Walters, Milford, CT, USA) was used, and measurements were expressed relative to a deuterated internal standard by multiple reaction monitoring using the transition 583>441 for MitoQ and 586>444 for d3-MitoQ. The results were analyzed using MassLynx software.

### Experiments in the chicken embryo

#### Egg incubation and MitoQ treatment

Fertilized Bovan Brown chicken eggs were obtained from Medeggs (Norfolk, UK). This supplier delivers fertilized eggs in batches from a single day’s laying, meaning that any egg used in the present program of work came from a different hen. Eggs were weighed and randomly chosen for incubation under normoxia (21% O_2_) or hypoxia (14 ± 0.5% O_2_) from the start of incubation period (incubator models 75-A and M-240, Masalles, Barcelona, Spain). All eggs were incubated at 37.9°C, 45% humidity and were automatically rotated hourly. Oxygen, temperature, and humidity were monitored continuously (oxygen probe: DD103 DrDAQ Oxygen Sensor, Pico Technology, St. Neots, UK), as described in detail previously (*36*, *37*). On day 13 of incubation (term is 21 days), a 1-mm hole was drilled in the shell above the air sac, allowing daily administration (between days 13 and 18) of 100 μl of vehicle (sterile water, Norbrook Laboratories, Carlisle, UK) or MitoQ (0.2 mg kg^−1^ day^−1^) onto the chorioallantoic membrane and hence into the embryonic circulation (*36*, *37*). On day 19, a blood sample was taken from the chorioallantoic artery (0.5-ml insulin syringe, BD Medical, Oxford, UK) for the measurement of hematocrit (140,000*g*, Micro-Hematocrit Centrifuge, Hawksley, Lancing, UK) (*36*, *37*). The embryo was then sacrificed via cervical transection, blotted, and weighed.

#### Langendorff isolated heart preparation

In the first cohort of embryos, hearts were dissected and immediately placed in ice-cold Krebs-Henseleit buffer (118.5 mM NaCl, 25 mM NaHCO_3_, 4.7 mM KCl, 1.2 mM MgSO_4_.7H_2_O, 1.2 mM KH_2_PO_4_, 1.25 mM CaCl_2_, 10 mM d-glucose, all from Sigma-Aldrich, Gillingham, UK) before being mounted onto the apparatus. Retrograde heart perfusion was performed via the aorta using Krebs solution at 38°C, oxygenated with 95% O_2_ and at a constant pressure corresponding to 2.66 kPa, the reported blood pressure of a day 19 chicken embryo (*36*, *38*). A small incision was made in the left atrium, and a balloon attached via a catheter to a pressure transducer (Argon Medical Devices, Plano, TX, USA) was inserted into the left ventricle. The balloon was filled with exactly 30 μl of water using a Hamilton syringe, which reproduces physiological basal measurements of left ventricular end diastolic pressure (LVEDP) in normoxic control embryos (*36*–*39*). After a period of stabilization, baseline measurement of LVDP was calculated by subtracting LVEDP from left ventricular systolic pressure.

#### In vitro wire myography in the chicken embryo

The left cranial tibial artery was dissected under a dissecting microscope (Stemi 2000, Zeiss, UK) from the same embryos used for the analysis of cardiac function via the Langendorff preparation at day 19 of incubation. Arterial segments (2 mm) were threaded with two pieces of stainless steel wire (40 μm diameter) and secured in a four-chamber microvascular myograph (Multi Wire Myograph System 610M; DMT, Aarhus, Denmark) (*36*, *39*). The myograph chambers were filled with Krebs-Henseleit buffer (118.5 mM NaCl, 25 mM NaHCO_3_, 4.7 mM KCl, 1.2 mM MgSO_4_.7H_2_O, 1.2 mM KH_2_PO_4_, 2.5 mM CaCl_2_, 2.8 mM d-glucose, all from Sigma-Aldrich, Gillingham, UK), which was aerated with 95% O_2_, 5% CO_2_, and the vessels were warmed to 37°C. Data were recorded using PowerLab and LabChart software (LabChart Pro 7.0, PowerLab 8/30; AD Instruments, Chalgrove, UK), and vessels were normalized using the DMT normalization module to 2.66 kPa. The vascular responsiveness to ACh (10^−9^ to 10^−4^ M) was assessed following preconstriction with a submaximal dose of potassium (to between 50 and 85% of their maximal constriction to 125 mM K^+^). ACh curves were also performed with a separate cohort of embryos, which were treated with only the carrier of MitoQ (dTPP-CD, 1.5 × 10^−9^ moles/day).

#### Cardiac mitochondrial respirometry

Hearts from a second cohort of embryos were immediately placed in ice-cold BIOPS solution, and left ventricular tissue was dissected into small pieces and incubated with saponin (50 μg μl^−1^, Sigma-Aldrich, Gillingham, UK). The tissue was then washed in ice-cold MiR05 solution, and 1 mg of tissue was transferred to a Clarke-type oxygen electrode (MT200, Strathkelvin Instruments, Motherwell, UK) in duplicate. Using Hamilton syringes, saturating concentrations of malate (2 mM) and glutamate (10 mM) were added, followed by adenosine diphosphate (ADP; 5 mM; all reagents from Sigma-Aldrich, Gillingham, UK). The respiratory control ratio was calculated by dividing complex I–mediated oxygen consumption in the presence of ADP by oxygen consumption in the presence of malate and glutamate only.

#### Cardiac stereology

A third cohort of embryos was heavily anesthetized (0.2 ml of sodium pentobarbital, I.P.; Pentoject, Animalcare Ltd., York, UK). A small incision was made in the right atrium, and the embryo was perfusion-fixed via the left ventricle by circulating a solution of heparinized saline [100 IU/ml heparin in 0.9% (w/v) NaCl, Animalcare Ltd., York, UK] followed by 10% paraformaldehyde (PFA) (Formal Fixx, Neutral Buffered Formalin, ThermoFisher Scientific, Loughborough, UK) at a constant pressure of 2.66 kPa. The hearts were immersed in 10% PFA for 24 hours before being transferred to phosphate-buffered saline. The hearts were sliced coronally into 1-mm-thick segments using a matrix (acrylic mouse heart slicer matrix, Zivic Instruments, Pittsburgh, PA, USA) and microtome blades (MX355 Premier, ThermoFisher Scientific, Loughborough, UK). The slices were arranged in order, apex side up and photographed. Using ImageJ software (version 1.46, National Institutes of Health, Bethesda, MD, USA), a 1-mm^2^ point grid was drawn over all cardiac sections and the number of points falling on left ventricular wall and left ventricular lumen was counted. This was converted into estimated left ventricular wall and lumen volumes using Cavalieri’s principle (*40*).

#### Measurement of MitoQ content in tissue with mass spectrometry

Embryonic hearts from a fourth cohort of all treatment groups were snap-frozen in liquid nitrogen at postmortem and stored at −80°C until analysis for levels of MitoQ. Frozen tissues were homogenized in 50 mM tris buffer (pH 7) and extracted with acetonitrile and evaporation. The extracts were reconstituted and the MitoQ contents of the tissue were measured using LC-MS/MS (*22*), as for the sheep tissues.

#### Mitochondria-targeted ratiometric mass spectrometry

On day 19, a fifth cohort of embryos from control and hypoxic groups with or without MitoQ was treated with 20 nmol MitoB dissolved in sterile water. The ratio of MitoP to MitoB is a retrospective measure of in vivo mitochondria-derived oxidative stress at the time of injection (*22*). In the hypoxic groups, MitoB was administered under hypoxic conditions within a transfer box to ensure that levels of in vivo oxidative stress measured represented the response to stable chronic hypoxia. Postmortem (under hypoxic conditions where appropriate) was performed exactly 1 hour after dosing. Tissues were isolated and snap-frozen in liquid nitrogen until analysis. Frozen tissues were homogenized in 50 mM tris buffer (pH 7) and extracted with acetonitrile and evaporation. The extracts were reconstituted, and the MitoP and MitoB contents of the tissue were measured using LC-MS/MS (*22*).

### Statistical analyses

The dataset for each comparison was checked for normality using the Shapiro-Wilk test, and if normality failed, data were transformed to a normal distribution before proceeding. All data are expressed as means ± SEM. Data were analyzed using two-way analysis of variance (ANOVA) or three-way ANOVA with and without repeated measures, as appropriate, using SigmaStat (version 3.5, Systat Software, San Jose, CA, USA) or SPSS (version 23, IBM SPSS Statistics, IL, USA). Please see table and figure legends for details. Where a significant interaction was observed between factors (indicated as H*Q on the graphs), the outcomes of a post hoc Tukey or Student’s *t* test are displayed. Where there was no significant interaction, any significant main effects are reported. For both main effects and interactions, * indicates a significant effect of hypoxia and † represents a significant effect of MitoQ. For all comparisons, significance was accepted when *P* < 0.05.

## Supplementary Material

abb1929_SM.pdf

## SUPPLEMENTARY MATERIALS

Supplementary material for this article is available at http://advances.sciencemag.org/cgi/content/full/6/34/eabb1929/DC1
