# Supplementary material for: Translatable mitochondria-targeted protection against programmed cardiovascular dysfunction
Source: Sci Adv. 2020 Aug 19;6(34):eabb1929. doi: 10.1126/sciadv.abb1929 (PMC7438086; doi:10.1126/sciadv.abb1929)
Supplement: abb1929_SM.pdf [file abb1929_SM.pdf]

Science Advances
AAAS

advances.sciencemag.org/cgi/content/full/6/34/eabb1929/DC1

Supplementary Materials for

Translatable mitochondria-targeted protection against programmed cardiovascular dysfunction

K. J. Botting, K. L. Skeffington, Y. Niu, B. J. Allison, K. L. Brain, N. Itani, C. Beck, A. Logan, A. J. Murray, M. P. Murphy, D. A. Giussani*

*Corresponding author. Email: dag26@cam.ac.uk

Published 19 August 2020, *Sci. Adv.* **6**, eabb1929 (2020)
DOI: 10.1126/sciadv.abb1929

This PDF file includes:

Tables S1 and S2
Fig. S1

SUPPLEMENTARY MATERIAL

Table S1. Blood gas status

A. Acute hypoxia – Fetal and maternal arterial blood gas measurements

			Normoxia			Acute hypoxia			Recovery	
			N0	N45	N75	H5	H15	H30	R30	R60
pH	Fetal CA	Saline	7.39 ± 0.01	7.37 ± 0.01	7.37 ± 0.01	7.33 ± 0.01*	7.29 ± 0.01*	7.25 ± 0.02*	7.28 ± 0.02*	7.32 ± 0.01*
		MitoQ	7.36 ± 0.01	7.36 ± 0.01	7.35 ± 0.01	7.33 ± 0.01*	7.29 ± 0.01*	7.28 ± 0.01*	7.31 ± 0.01*	7.33 ± 0.01*
	Fetal FA	Saline	7.38 ± 0.01	7.37 ± 0.01	7.37 ± 0.01	7.34 ± 0.01*	7.29 ± 0.01*	7.24 ± 0.02*	7.29 ± 0.01*	7.31 ± 0.01*
		MitoQ	7.37 ± 0.01	7.35 ± 0.01	7.35 ± 0.01	7.34 ± 0.01*	7.30 ± 0.01*	7.26 ± 0.01*	7.31 ± 0.01*	7.32 ± 0.01*
	Maternal FA	Saline	7.51 ± 0.01	7.52 ± 0.01	7.51 ± 0.01	7.45 ± 0.01	7.44 ± 0.02*	7.44 ± 0.02*	7.48 ± 0.02	7.50 ± 0.01
		MitoQ	7.49 ± 0.01	7.49 ± 0.01	7.49 ± 0.01	7.47 ± 0.01	7.44 ± 0.01*	7.47 ± 0.01*	7.50 ± 0.01	7.50 ± 0.01
$PaCO_2$ (mmHg)	Fetal CA	Saline	49.30 ± 1.38	51.60 ± 1.29	50.80 ± 1.53	54.20 ± 0.86	53.80 ± 1.77	53.40 ± 2.86	50.40 ± 1.44	50.60 ± 1.96
		MitoQ	52.00 ± 0.65	51.80 ± 1.32	52.60 ± 0.87	55.20 ± 1.11	55.60 ± 0.51	54.80 ± 2.40	51.60 ± 1.69	53.00 ± 2.02
	Fetal FA	Saline	52.83 ± 0.59	53.00 ± 1.41	54.67 ± 1.12	56.20 ± 1.02	57.67 ± 1.33*	58.50 ± 1.48*	52.00 ± 1.41	54.33 ± 1.96
		MitoQ	54.08 ± 0.66	56.00 ± 1.41	55.67 ± 0.84	56.50 ± 0.62	60.00 ± 1.13*	57.17 ± 1.56*	53.33 ± 1.36	56.50 ± 1.67
	Maternal FA	Saline	34.42 ± 0.62	33.83 ± 0.70	35.67 ± 0.67	39.40 ± 1.72	40.33 ± 0.61	39.83 ± 0.79	36.83 ± 1.45	35.50 ± 1.18
		MitoQ	36.33 ± 0.77	35.83 ± 0.54	37.00 ± 1.00	39.50 ± 0.67	41.33 ± 0.80	39.17 ± 1.40	36.17 ± 1.28	37.00 ± 1.46
PaO_2 (mmHg)	Fetal CA	Saline	24.00 ± 1.52	22.40 ± 1.81	22.20 ± 2.08	14.60 ± 0.93*	13.00 ± 0.58*	12.80 ± 0.49*	25.60 ± 1.36	23.80 ± 1.43
		MitoQ	24.10 ± 1.07	22.00 ± 2.68	24.20 ± 1.96	14.00 ± 0.89*	13.75 ± 0.48*	13.60 ± 0.24*	25.00 ± 1.47	24.80 ± 2.80
	Fetal FA	Saline	20.25 ± 1.13	19.50 ± 1.78	19.00 ± 2.02	10.50 ± 0.81*	11.00 ± 0.82*	10.83 ± 0.48*	22.17 ± 1.47	20.50 ± 0.96
		MitoQ	20.42 ± 1.50	19.67 ± 1.33	19.83 ± 1.14	11.17 ± 1.08*	10.17 ± 0.75*	11.00 ± 0.52*	20.83 ± 1.58	18.67 ± 1.17
	Maternal FA	Saline	99.67 ± 3.85	100.67 ± 2.70	97.50 ± 3.92	41.17 ± 7.54*	33.58 ± 2.15*	31.00 ± 2.13*	97.00 ± 3.84	100.67 ± 6.58
		MitoQ	101.33 ± 1.89	114.33 ± 7.66	103.00 ± 3.94	35.80 ± 3.43*	34.50 ± 2.54*	33.83 ± 2.65*	110.33 ± 4.92	105.00 ± 3.30
SatHb (%)	Fetal CA	Saline	69.18 ± 0.80	65.05 ± 4.36	65.18 ± 2.66	34.18 ± 4.40*	29.63 ± 2.72*	23.83 ± 2.80*	72.10 ± 2.08	68.78 ± 2.44
		MitoQ	74.38 ± 5.91	76.68 ± 9.45	70.98 ± 4.25	33.57 ± 4.83*	26.20 ± 0.83*	25.13 ± 3.61*	67.88 ± 2.16	71.13 ± 4.80
	Fetal FA	Saline	62.71 ± 2.93	56.08 ± 3.80	58.12 ± 3.53	24.54 ± 2.41*	19.74 ± 2.50*	19.92 ± 3.32*	62.80 ± 3.01	57.70 ± 2.55*
		MitoQ	62.08 ± 2.40	59.02 ± 0.77	59.71 ± 1.62	25.56 ± 3.09*	21.36 ± 2.74*	20.46 ± 2.36*	59.78 ± 0.68	52.22 ± 1.90*
	Maternal FA	Saline	103.88 ± 0.54	104.16 ± 0.67	103.38 ± 0.98	56.74 ± 5.23*	54.68 ± 6.07*	49.12 ± 5.61*	103.52 ± 1.10	102.08 ± 1.27
		MitoQ	103.62 ± 0.71	104.00 ± 0.54	103.62 ± 0.79	63.04 ± 5.64*	59.84 ± 7.27*	56.08 ± 4.29*	104.32 ± 0.48	103.90 ± 0.83

Hb ($g.dL^{-1}$)	Fetal CA	Saline	9.36 ± 0.34	9.18 ± 0.40	8.78 ± 0.24	10.50 ± 0.31	10.15 ± 0.32	10.43 ± 0.13	8.98 ± 0.39	8.93 ± 0.41
		MitoQ	8.39 ± 0.49	8.40 ± 0.43	8.33 ± 0.31	9.25 ± 0.34	9.38 ± 0.23	9.28 ± 0.26	8.00 ± 0.23	7.95 ± 0.41
	Fetal FA	Saline	9.75 ± 0.50	9.80 ± 0.44	9.90 ± 0.45	10.68 ± 0.28	10.56 ± 0.39	10.55 ± 0.23	9.24 ± 0.48	9.38 ± 0.47
		MitoQ	8.61 ± 0.43†	8.54 ± 0.34†	8.54 ± 0.32†	9.42 ± 0.25†	9.30 ± 0.33†	9.30 ± 0.22†	8.26 ± 0.29†	8.40 ± 0.27†
	Maternal FA	Saline	10.57 ± 0.17	10.18 ± 0.30	10.38 ± 0.20	12.60 ± 0.37	12.72 ± 0.26*	13.56 ± 0.34*	10.40 ± 0.32	9.72 ± 0.45
		MitoQ	10.37 ± 0.44	10.46 ± 0.44	10.48 ± 0.39	11.76 ± 0.39	12.70 ± 0.35*	12.22 ± 0.37	10.78 ± 0.58	10.68 ± 0.43

B. Chronic hypoxia - maternal arterial blood gas measurements

			Gestational age (d)							
			105	106	110	115	120	125	130	135
pHa	Normoxia	Saline	7.50 ± 0.01	7.51 ± 0.01	7.50 ± 0.01	7.49 ± 0.01	7.49 ± 0.01	7.49 ± 0.01	7.48 ± 0.01	7.50 ± 0.01
		MitoQ	7.50 ± 0.01	7.49 ± 0.01	7.49 ± 0.01	7.49 ± 0.01	7.49 ± 0.01	7.49 ± 0.01	7.49 ± 0.01	7.48 ± 0.01†
	Hypoxia	Saline	7.50 ± 0.01	7.54 ± 0.01*	7.51 ± 0.01	7.51 ± 0.01	7.51 ± 0.01	7.50 ± 0.01	7.51 ± 0.01	7.50 ± 0.01
		MitoQ	7.50 ± 0.01	7.52 ± 0.01*	7.52 ± 0.01	7.50 ± 0.01	7.50 ± 0.01	7.50 ± 0.01	7.50 ± 0.01	7.49 ± 0.01†
$PaCO_2$ (mmHg)	Normoxia	Saline	34.37 ± 0.56	35.78 ± 0.69	35.24 ± 0.81	37.00 ± 1.26	38.00 ± 1.40	36.69 ± 1.29	38.09 ± 1.53	38.07 ± 1.04
		MitoQ	34.11 ± 0.40	31.33 ± 1.71	33.93 ± 0.31	33.70 ± 0.31	34.34 ± 0.37	34.28 ± 0.31	34.41 ± 0.39	34.97 ± 0.41
	Hypoxia	Saline	33.66 ± 0.41	27.95 ± 0.56*	27.96 ± 0.28*	29.63 ± 0.35*	29.64 ± 0.37*	30.41 ± 0.77*	30.24 ± 0.36*	29.84 ± 0.40*
		MitoQ	33.37 ± 0.30	29.10 ± 1.05*	29.04 ± 0.21*	30.17 ± 0.26*	30.03 ± 0.36*	30.10 ± 0.31*	30.12 ± 0.40*	30.44 ± 0.39*
PaO_2 (mmHg)	Normoxia	Saline	101.77 ± 2.25	101.00 ± 2.31	107.28 ± 3.07	108.08 ± 1.86	105.89 ± 2.96	109.73 ± 4.85	102.11 ± 2.25	101.73 ± 2.86
		MitoQ	102.37 ± 1.45	123.60 ± 9.40†	107.50 ± 1.30	106.94 ± 1.64	106.88 ± 1.47	106.47 ± 1.65	106.57 ± 1.80	101.45 ± 1.54
	Hypoxia	Saline	105.59 ± 1.78	51.67 ± 2.02*	48.59 ± 1.04*	46.24 ± 0.73*	46.96 ± 0.66*	46.44 ± 0.62*	46.77 ± 0.75*	45.22 ± 0.72*
		MitoQ	105.33 ± 1.74	62.95 ± 1.63*†	52.08 ± 0.63*	47.83 ± 0.39*	47.53 ± 0.46*	48.06 ± 0.56*	47.23 ± 0.49*	48.01 ± 0.46*
Sat Hb (%)	Normoxia	Saline	103.08 ± 0.32	103.05 ± 0.50	103.70 ± 0.58	103.74 ± 0.47	104.62 ± 0.54	103.88 ± 0.53	104.37 ± 0.71	103.89 ± 0.62
		MitoQ	102.03 ± 0.32	103.42 ± 0.58	102.49 ± 0.21	102.43 ± 0.31	102.93 ± 0.17	102.83 ± 0.26	102.64 ± 0.24	101.93 ± 0.34
	Hypoxia	Saline	101.45 ± 1.48	85.74 ± 2.03*	81.67 ± 1.04*	80.21 ± 0.99*	80.30 ± 1.03*	79.34 ± 1.35*	80.98 ± 0.87*	78.58 ± 1.11*
		MitoQ	101.70 ± 1.42	95.34 ± 0.90*	86.71 ± 0.67*	83.28 ± 0.69*	82.51 ± 0.83*	82.04 ± 0.88*	82.55 ± 1.04*	82.89 ± 0.94*
Hb ($g.dL^{-1}$)	Normoxia	Saline	11.23 ± 0.18	11.02 ± 0.30	10.26 ± 0.34	9.54 ± 0.33	9.87 ± 0.37	10.08 ± 0.35	9.89 ± 0.32	9.74 ± 0.29
		MitoQ	10.55 ± 0.15	10.67 ± 0.35	10.11 ± 0.13	9.72 ± 0.10	9.54 ± 0.09	9.53 ± 0.13	9.94 ± 0.11	9.73 ± 0.10
	Hypoxia	Saline	11.44 ± 0.16	11.88 ± 0.32*	11.51 ± 0.12*	11.69 ± 0.11*	11.87 ± 0.14*	12.25 ± 0.12*	12.52 ± 0.13*	12.70 ± 0.12*
		MitoQ	11.00 ± 0.18	11.47 ± 0.33*	11.13 ± 0.13*	10.92 ± 0.11*	11.19 ± 0.15*	11.65 ± 0.10*	11.69 ± 0.10*	11.98 ± 0.14*

Values are mean±SEM for maternal and fetal arterial pH, PCO_2, PO_2, hemoglobin saturation with oxygen (SatHb) and hemoglobin concentration (Hb) in blood samples taken during the acute hypoxia protocol (A) or with advancing gestational age (B) in normoxic or hypoxic pregnancy with and without maternal treatment with MitoQ. For (A), blood samples were taken at 0, 45 and 75 min of baseline normoxia (N), 5, 15 and 30 min of acute hypoxia (H) and after 30 and 60 min of recovery (R). For (B), blood samples were taken at 105 and 106 days of gestation (before and after onset of MitoQ treatment) and at 5-day intervals until 135 days of gestation. The effects of Hypoxia and MitoQ treatment were determined by two-way ANOVA with repeated measures where appropriate. * indicates a significant effect of Hypoxia; †indicates a significant effect of MitoQ.

Table S2. Ovine fetal and adult offspring biometry

	**Fetal offspring**				**Adult offspring**			
	Normoxic	Hypoxic	Hypoxic +MitoQ	Normoxic +MitoQ	Normoxic	Hypoxic	Hypoxic +MitoQ	Normoxic +MitoQ
Brain								
Absolute (g)	49.9 ± 0.9	38.8 ± 2.3*	43.5 ± 1.7*†	48.1 ± 1.3†	84.5 ± 1.9	89.6 ± 2.0	91.5 ± 3.6	86.7 ± 2.6
Relative ($g.kg^{-1}$)	12.6 ± 0.7	14.9 ± 1.0*	13.3 ± 0.6*	11.6 ± 0.4	3.4 ± 0.2	3.0 ± 0.2	2.9 ± 0.2	3.1 ± 0.1
Heart								
Absolute (g)	34.4 ± 3.5	25.4 ± 2.7*	30.2 ± 2.0*	37.0 ± 2.5	215.0 ± 11.9	239.5 ± 10.7	226.5 ± 12.8	212.2 ± 12.3
Relative ($g.kg^{-1}$)	9.8 ± 0.8	9.5 ± 0.3	9.1 ± 0.3	8.8 ± 0.4	8.6 ± 0.3	8.0 ± 0.4	7.1 ± 0.4†	7.6 ± 0.4†
Liver								
Absolute (g)	77.1 ± 6.5	56.6 ± 7.2*	68.8 ± 3.4*†	102.1 ± 5.6†	387.4 ± 19.6	422.2 ± 32.2*	466.1 ± 28.5*	374.7 ± 21.6
Relative ($g.kg^{-1}$)	21.8 ± 0.9	21.0 ± 1.8	20.9 ± 0.6	24.4 ± 1.1	15.5 ± 1.1	14.2 ± 1.3	14.5 ± 0.6	13.8 ± 0.9

Values are mean±SEM for fetal and adult absolute organ weights and organ weights relative to body weight in normoxic or hypoxic pregnancy with and without maternal treatment with MitoQ. The effects of Hypoxia and MitoQ treatment were determined by two-way ANOVA. * indicates a significant effect of Hypoxia. † indicates a significant effect of MitoQ.

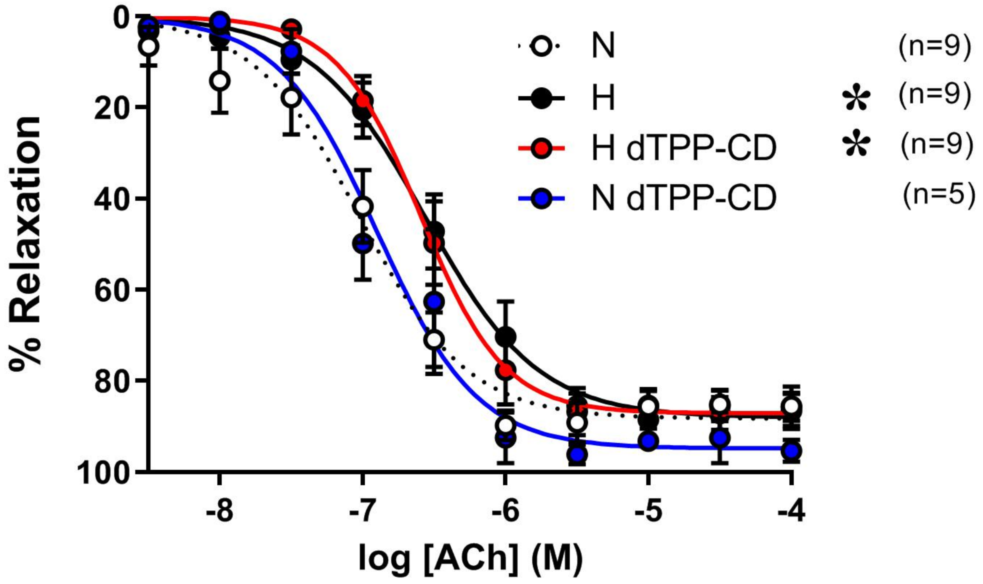

Figure S1. Effect of chronic hypoxia and MitoQ carrier treatment in the chicken embryo. Values are the mean±SEM of the concentration response curves using *in vitro* wire myography to increasing doses of acetylcholine (ACh) in pre-constricted femoral vessels isolated from chicken embryos of untreated normoxic (N, white) or hypoxic (H, black) incubation, or from normoxic (N dTPP-CD, blue) or hypoxic (H dTPP-CD, red) incubation treated with the MitoQ carrier dTPP-CD. The effect of Hypoxia and MitoQ treatment were determined by two-way ANOVA with repeated measures. * indicates a significant effect of Hypoxia using area above the curve.
